# 2-[(5,7-Dibromo­quinolin-8-yl)­oxy]-*N*-(2-meth­oxy­phen­yl)acetamide

**DOI:** 10.1107/S1600536810048312

**Published:** 2010-11-24

**Authors:** Yong-Hong Wen, Hong-Qing Qin, Hui-Ling Wen

**Affiliations:** a College of Chemistry and Molecular Engineering, Qingdao University of Science and Technology, Qingdao 266042, People’s Republic of China

## Abstract

In the title compound, C_18_H_14_Br_2_N_2_O_3_, an intra­molecular N—H⋯N hydrogen bond forms an eight-membered ring. The dihedral angle between the planes of the quinoline system and the benzene ring is 41.69 (1)°. The crystal packing is stabilized by inter­molecular C—H⋯O hydrogen bonds and short Br⋯O inter­actions [3.0079 (19) Å].

## Related literature

The structure of *N*,*N*-dicyclo­hexyl-2-(5,7-dibromo­quinolin-8-yl­oxy)acetamide has been reported by Liu *et al.* (2007 ▶). For bond-length data, see: Allen *et al.* (1987 ▶). For applications of 8-hy­droxy­quinoline and its derivatives, see: Bratzel *et al.* (1972 ▶). Some 8-hy­droxy­quinoline derivatives and their trans­ition metal complexes exhibit anti­bacterial activity, see: Patel & Patel (1999 ▶).
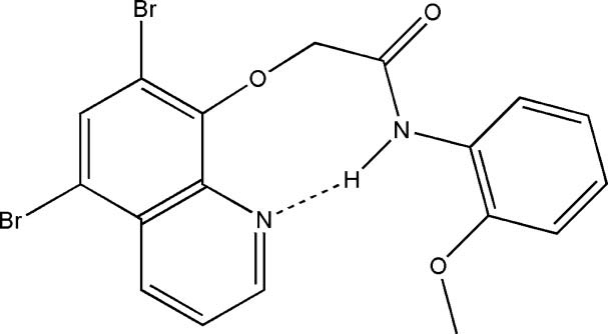


## Experimental

### 

#### Crystal data

C_18_H_14_Br_2_N_2_O_3_
*M* *_r_* = 466.13
Monoclinic, 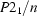

*a* = 8.7570 (18) Å
*b* = 8.7279 (17) Å
*c* = 22.372 (5) Å
β = 98.04 (3)°
*V* = 1693.1 (6) Å^3^
*Z* = 4
Mo *K*α radiation
μ = 4.81 mm^−1^
*T* = 293 K
0.06 × 0.02 × 0.02 mm

#### Data collection

Bruker SMART CCD area-detector diffractometer
Absorption correction: multi-scan (*SADABS*; Sheldrick, 1996 ▶) *T*_min_ = 0.761, *T*_max_ = 0.910
12864 measured reflections
4027 independent reflections
3316 reflections with *I* > 2σ(*I*)
*R*_int_ = 0.057

#### Refinement

*R*[*F* ^2^ > 2σ(*F* ^2^)] = 0.044
*wR*(*F* ^2^) = 0.093
*S* = 1.06
4027 reflections
231 parameters
1 restraint
H atoms treated by a mixture of independent and constrained refinement
Δρ_max_ = 0.83 e Å^−3^
Δρ_min_ = −0.71 e Å^−3^

### 

Data collection: *SMART* (Bruker, 2001 ▶); cell refinement: *SAINT* (Bruker, 2001 ▶); data reduction: *SAINT*; program(s) used to solve structure: *SHELXTL* (Sheldrick, 2008 ▶); program(s) used to refine structure: *SHELXTL*; molecular graphics: *SHELXTL*; software used to prepare material for publication: *SHELXTL*.

## Supplementary Material

Crystal structure: contains datablocks I, global. DOI: 10.1107/S1600536810048312/hg2753sup1.cif

Structure factors: contains datablocks I. DOI: 10.1107/S1600536810048312/hg2753Isup2.hkl

Additional supplementary materials:  crystallographic information; 3D view; checkCIF report

## Figures and Tables

**Table 1:** Hydrogen-bond geometry (Å, °)

*D*—H⋯*A*	*D*—H	H⋯*A*	*D*⋯*A*	*D*—H⋯*A*
N2—H2*A*⋯N1	0.90 (1)	2.24 (1)	3.065 (3)	153 (1)
C18—H18*C*⋯O2^i^	0.96	2.53	3.342 (3)	142

Symmetry code: (i) 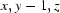.

**Fig. 1.**
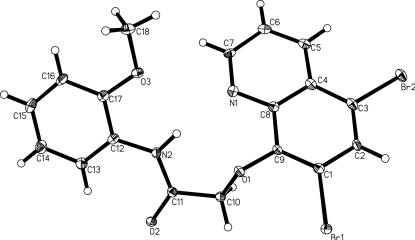
The molecular structure of (I), with atom labels and 30% probability displacement ellipsoids.

**Fig. 2.**
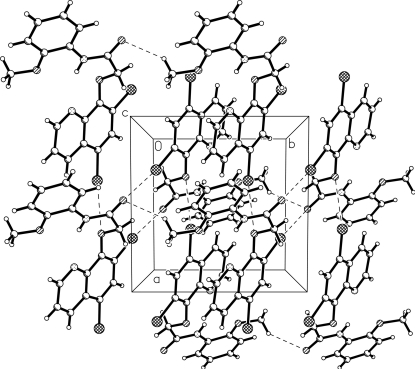
The packing diagram of (I), viewed down the c axis, showing the intermolecular hydrogen bonds (dashed lines).

## supplementary crystallographic information

### Comment

8-Hydroxyquinoline and its derivatives have found extensive application as analytical reagents, *e.g.* in absorption spectrophotometry, fluorimetry, solvent extraction and partition chromatography (Bratzel *et al.*, 1972). Some 8-hydroxyquinoline derivatives and their complexes with transition metals demonstrate antibacterial activity (Patel & Patel,1999). Recently, the structure of 5,7-dibromosubstituted 8-hydroxyquinolinate amide-type compound, namely *N*,*N*-dicyclohexyl-2-(5,7-dibromoquinolin-8-yloxy)acetamide, (II), has been reported (Liu *et al.*, 2007). Here, we have synthesized and carried out the structure determination of the title compound, (I), (Fig. 1).

All bond lengths in (I) are within normal ranges (Allen *et al.*, 1987) and comparable with those in the related compound (II). The sum of the angles around atoms N2 and C11 are 359.9° and 360.0°, respectively, implying a planar configuration. There is one intramolecular hydrogen bond, *viz.* N2—H2···N1 (Table 1), forming one larger eight-membered ring. The dihedral angle between the planes of the quinoline system and the benzene ring is 41.69 (1)°. The crystal packing is stabilized by intermolecular C18—H18C···O2 hydrogen bond (Table 1) and Br···O short-contact interactions.

### Experimental

To a solution of 5,7-dibromo-8-hydroxyquinoline (3.02 g, 10 mmol) in acetone (60 ml) were added 2-chloro-*N*-(4-methoxyphenyl)acetamide (2.0 g,10 mmol), K_2_CO_3_ (1.52 g, 11 mmol) and KI (0.5 g), and the resulting mixture was stirred at 333 K for 5 h. After cooling to room temperature, the mixture was washed three times with water and filtered. Colourless single crystals of (I) suitable for X-ray diffraction study were obtained by slow evaporation of an acetone solution over a period of 6 d.

### Refinement

H atoms were positioned geometrically, with C—H = 0.95–0.99 Å, respectively, and constrained to ride on their parent atoms, with *U*_iso_(H) = 1.2 *U*_eq_(C). The amide proton was refined freely, giving a N—H bond distance of 0.898 (9) Å.

### Crystal data

**Table:**

C_18_H_14_Br_2_N_2_O_3_	*F*(000) = 920
*M**_r_* = 466.13	*D*_x_ = 1.829 Mg m^−^^3^
Monoclinic, *P*2_1_/*n*	Mo *K*α radiation, λ = 0.71073 Å
Hall symbol: -P 2yn	Cell parameters from 4165 reflections
*a* = 8.7570 (18) Å	θ = 1.8–27.9°
*b* = 8.7279 (17) Å	µ = 4.81 mm^−^^1^
*c* = 22.372 (5) Å	*T* = 293 K
β = 98.04 (3)°	Column, colourless
*V* = 1693.1 (6) Å^3^	0.06 × 0.02 × 0.02 mm
*Z* = 4	

### Data collection

**Table:**

Bruker SMART CCD area-detector diffractometer	4027 independent reflections
Radiation source: fine-focus sealed tube	3316 reflections with *I* > 2σ(*I*)
graphite	*R*_int_ = 0.057
phi and ω scans	θ_max_ = 27.9°, θ_min_ = 1.8°
Absorption correction: multi-scan (*SADABS*; Sheldrick, 1996)	*h* = −11→11
*T*_min_ = 0.761, *T*_max_ = 0.910	*k* = −11→8
12864 measured reflections	*l* = −29→28

### Refinement

**Table:**

Refinement on *F*^2^	Primary atom site location: structure-invariant direct methods
Least-squares matrix: full	Secondary atom site location: difference Fourier map
*R*[*F*^2^ > 2σ(*F*^2^)] = 0.044	Hydrogen site location: inferred from neighbouring sites
*wR*(*F*^2^) = 0.093	H atoms treated by a mixture of independent and constrained refinement
*S* = 1.06	*w* = 1/[σ^2^(*F*_o_^2^) + (0.0337*P*)^2^ + 1.4595*P*] where *P* = (*F*_o_^2^ + 2*F*_c_^2^)/3
4027 reflections	(Δ/σ)_max_ = 0.005
231 parameters	Δρ_max_ = 0.83 e Å^−^^3^
1 restraint	Δρ_min_ = −0.71 e Å^−^^3^

### Special details

**Table:**

Geometry. All e.s.d.'s (except the e.s.d. in the dihedral angle between two l.s. planes) are estimated using the full covariance matrix. The cell e.s.d.'s are taken into account individually in the estimation of e.s.d.'s in distances, angles and torsion angles; correlations between e.s.d.'s in cell parameters are only used when they are defined by crystal symmetry. An approximate (isotropic) treatment of cell e.s.d.'s is used for estimating e.s.d.'s involving l.s. planes.
Refinement. Refinement of *F*^2^ against ALL reflections. The weighted *R*-factor *wR* and goodness of fit *S* are based on *F*^2^, conventional *R*-factors *R* are based on *F*, with *F* set to zero for negative *F*^2^. The threshold expression of *F*^2^ > σ(*F*^2^) is used only for calculating *R*-factors(gt) *etc*. and is not relevant to the choice of reflections for refinement. *R*-factors based on *F*^2^ are statistically about twice as large as those based on *F*, and *R*- factors based on ALL data will be even larger.

### Fractional atomic coordinates and isotropic or equivalent isotropic displacement parameters (Å^2^)

**Table:**

	*x*	*y*	*z*	*U*_iso_*/*U*_eq_	
Br1	0.77254 (3)	0.41089 (3)	1.064695 (11)	0.01979 (6)	
Br2	0.16180 (3)	0.18905 (3)	1.014627 (12)	0.02468 (7)	
O1	0.81081 (18)	0.22709 (18)	0.94999 (7)	0.0173 (4)	
O2	1.0293 (2)	0.39129 (19)	0.84558 (8)	0.0241 (4)	
O3	0.8399 (2)	−0.13107 (19)	0.82222 (8)	0.0262 (5)	
N2	0.9123 (2)	0.1556 (2)	0.84366 (9)	0.0171 (5)	
N1	0.5971 (2)	0.0662 (2)	0.87776 (9)	0.0190 (5)	
C1	0.6236 (3)	0.2953 (3)	1.01398 (11)	0.0171 (5)	
C2	0.4727 (3)	0.2851 (3)	1.02927 (11)	0.0180 (5)	
H2	0.4474	0.3365	1.0630	0.022*	
C3	0.3646 (3)	0.1998 (3)	0.99438 (11)	0.0189 (6)	
C4	0.3988 (3)	0.1219 (3)	0.94234 (11)	0.0168 (5)	
C5	0.2943 (3)	0.0290 (3)	0.90434 (11)	0.0203 (6)	
H5	0.1941	0.0150	0.9127	0.024*	
C6	0.3416 (3)	−0.0401 (3)	0.85523 (12)	0.0222 (6)	
H6	0.2736	−0.1005	0.8297	0.027*	
C7	0.4956 (3)	−0.0190 (3)	0.84358 (11)	0.0185 (6)	
H7	0.5262	−0.0676	0.8102	0.022*	
C8	0.5505 (3)	0.1348 (3)	0.92702 (11)	0.0167 (5)	
C9	0.6633 (3)	0.2237 (3)	0.96423 (10)	0.0144 (5)	
C10	0.8431 (3)	0.3518 (3)	0.91172 (11)	0.0199 (6)	
H10A	0.8976	0.4317	0.9362	0.024*	
H10B	0.7467	0.3946	0.8920	0.024*	
C11	0.9396 (3)	0.3002 (3)	0.86413 (11)	0.0167 (5)	
C12	0.9771 (3)	0.0828 (3)	0.79679 (11)	0.0171 (6)	
C13	1.0774 (3)	0.1524 (3)	0.76220 (11)	0.0219 (6)	
H13	1.1071	0.2536	0.7697	0.026*	
C14	1.1340 (3)	0.0712 (3)	0.71627 (12)	0.0267 (7)	
H14	1.2012	0.1188	0.6934	0.032*	
C15	1.0912 (3)	−0.0787 (3)	0.70449 (12)	0.0270 (7)	
H15	1.1279	−0.1317	0.6733	0.032*	
C16	0.9923 (3)	−0.1508 (3)	0.73961 (12)	0.0244 (6)	
H16	0.9645	−0.2526	0.7322	0.029*	
C17	0.9358 (3)	−0.0713 (3)	0.78527 (11)	0.0194 (6)	
C18	0.8150 (3)	−0.2923 (3)	0.82018 (13)	0.0284 (7)	
H18A	0.7705	−0.3216	0.7801	0.043*	
H18B	0.7460	−0.3198	0.8482	0.043*	
H18C	0.9116	−0.3443	0.8308	0.043*	
H2A	0.8405 (13)	0.106 (2)	0.8612 (8)	0.032 (8)*	

### Atomic displacement parameters (Å^2^)

**Table:**

	*U*^11^	*U*^22^	*U*^33^	*U*^12^	*U*^13^	*U*^23^
Br1	0.02090 (11)	0.02052 (12)	0.01835 (12)	−0.00516 (10)	0.00418 (10)	−0.00258 (10)
Br2	0.01560 (11)	0.02843 (13)	0.03130 (14)	−0.00014 (10)	0.00784 (10)	0.00388 (11)
O1	0.0136 (7)	0.0182 (8)	0.0208 (8)	−0.0006 (7)	0.0048 (7)	0.0022 (7)
O2	0.0284 (9)	0.0165 (8)	0.0304 (10)	−0.0091 (7)	0.0149 (8)	−0.0046 (7)
O3	0.0305 (9)	0.0132 (8)	0.0377 (10)	−0.0036 (7)	0.0139 (8)	−0.0035 (8)
N2	0.0194 (9)	0.0132 (9)	0.0200 (10)	0.0007 (8)	0.0076 (8)	0.0011 (8)
N1	0.0197 (10)	0.0200 (10)	0.0168 (10)	0.0022 (8)	0.0012 (9)	0.0018 (8)
C1	0.0177 (11)	0.0161 (11)	0.0175 (12)	0.0000 (9)	0.0019 (9)	0.0037 (9)
C2	0.0178 (11)	0.0173 (11)	0.0209 (12)	0.0003 (9)	0.0096 (10)	0.0026 (10)
C3	0.0159 (11)	0.0180 (11)	0.0232 (12)	0.0030 (9)	0.0044 (10)	0.0098 (10)
C4	0.0151 (10)	0.0173 (11)	0.0172 (12)	0.0031 (9)	−0.0006 (9)	0.0085 (10)
C5	0.0160 (11)	0.0179 (11)	0.0261 (13)	−0.0019 (10)	0.0000 (10)	0.0044 (10)
C6	0.0220 (12)	0.0176 (12)	0.0250 (13)	−0.0035 (10)	−0.0038 (11)	0.0029 (10)
C7	0.0252 (12)	0.0149 (11)	0.0143 (11)	−0.0027 (10)	−0.0008 (10)	−0.0010 (10)
C8	0.0202 (11)	0.0138 (10)	0.0161 (11)	0.0007 (9)	0.0025 (10)	0.0048 (9)
C9	0.0117 (10)	0.0141 (11)	0.0175 (11)	0.0006 (9)	0.0030 (9)	0.0035 (9)
C10	0.0242 (12)	0.0127 (11)	0.0247 (12)	−0.0028 (10)	0.0099 (10)	−0.0012 (10)
C11	0.0152 (10)	0.0158 (11)	0.0191 (12)	0.0012 (9)	0.0024 (9)	0.0012 (10)
C12	0.0200 (11)	0.0160 (11)	0.0138 (11)	0.0025 (9)	−0.0023 (10)	0.0001 (9)
C13	0.0245 (12)	0.0184 (12)	0.0230 (13)	0.0010 (10)	0.0046 (11)	0.0030 (10)
C14	0.0328 (14)	0.0299 (14)	0.0193 (13)	0.0003 (12)	0.0105 (11)	0.0010 (11)
C15	0.0327 (14)	0.0300 (14)	0.0184 (13)	0.0084 (12)	0.0040 (11)	−0.0057 (11)
C16	0.0288 (13)	0.0183 (12)	0.0261 (13)	0.0028 (11)	0.0031 (11)	−0.0082 (11)
C17	0.0188 (11)	0.0197 (12)	0.0188 (12)	0.0018 (10)	−0.0009 (10)	−0.0010 (10)
C18	0.0332 (14)	0.0164 (12)	0.0357 (15)	−0.0043 (11)	0.0055 (13)	−0.0017 (11)

### Geometric parameters (Å, °)

**Table:**

Br1—C1	1.895 (2)	C6—C7	1.420 (4)
Br2—C3	1.896 (2)	C6—H6	0.9300
O1—C9	1.374 (3)	C7—H7	0.9300
O1—C10	1.437 (3)	C8—C9	1.429 (3)
O2—C11	1.230 (3)	C10—C11	1.518 (3)
O3—C17	1.362 (3)	C10—H10A	0.9700
O3—C18	1.424 (3)	C10—H10B	0.9700
N2—C11	1.352 (3)	C12—C13	1.389 (4)
N2—C12	1.411 (3)	C12—C17	1.407 (3)
N2—H2A	0.898 (9)	C13—C14	1.395 (4)
N1—C7	1.318 (3)	C13—H13	0.9300
N1—C8	1.366 (3)	C14—C15	1.376 (4)
C1—C9	1.363 (3)	C14—H14	0.9300
C1—C2	1.414 (3)	C15—C16	1.398 (4)
C2—C3	1.361 (3)	C15—H15	0.9300
C2—H2	0.9300	C16—C17	1.383 (4)
C3—C4	1.416 (3)	C16—H16	0.9300
C4—C5	1.415 (3)	C18—H18A	0.9600
C4—C8	1.421 (3)	C18—H18B	0.9600
C5—C6	1.368 (4)	C18—H18C	0.9600
C5—H5	0.9300		
			
C9—O1—C10	115.11 (17)	O1—C10—C11	111.60 (19)
C17—O3—C18	117.6 (2)	O1—C10—H10A	109.3
C11—N2—C12	127.0 (2)	C11—C10—H10A	109.3
C11—N2—H2A	113.8 (14)	O1—C10—H10B	109.3
C12—N2—H2A	119.1 (14)	C11—C10—H10B	109.3
C7—N1—C8	117.5 (2)	H10A—C10—H10B	108.0
C9—C1—C2	121.5 (2)	O2—C11—N2	125.5 (2)
C9—C1—Br1	120.03 (18)	O2—C11—C10	119.3 (2)
C2—C1—Br1	118.47 (18)	N2—C11—C10	115.2 (2)
C3—C2—C1	119.6 (2)	C13—C12—C17	118.8 (2)
C3—C2—H2	120.2	C13—C12—N2	124.7 (2)
C1—C2—H2	120.2	C17—C12—N2	116.5 (2)
C2—C3—C4	121.6 (2)	C12—C13—C14	120.4 (2)
C2—C3—Br2	119.32 (19)	C12—C13—H13	119.8
C4—C3—Br2	119.06 (17)	C14—C13—H13	119.8
C5—C4—C3	125.1 (2)	C15—C14—C13	120.6 (3)
C5—C4—C8	116.7 (2)	C15—C14—H14	119.7
C3—C4—C8	118.2 (2)	C13—C14—H14	119.7
C6—C5—C4	119.5 (2)	C14—C15—C16	119.6 (3)
C6—C5—H5	120.3	C14—C15—H15	120.2
C4—C5—H5	120.3	C16—C15—H15	120.2
C5—C6—C7	119.5 (2)	C17—C16—C15	120.2 (2)
C5—C6—H6	120.3	C17—C16—H16	119.9
C7—C6—H6	120.3	C15—C16—H16	119.9
N1—C7—C6	123.2 (2)	O3—C17—C16	124.8 (2)
N1—C7—H7	118.4	O3—C17—C12	114.8 (2)
C6—C7—H7	118.4	C16—C17—C12	120.4 (2)
N1—C8—C4	123.7 (2)	O3—C18—H18A	109.5
N1—C8—C9	116.6 (2)	O3—C18—H18B	109.5
C4—C8—C9	119.7 (2)	H18A—C18—H18B	109.5
C1—C9—O1	122.3 (2)	O3—C18—H18C	109.5
C1—C9—C8	119.4 (2)	H18A—C18—H18C	109.5
O1—C9—C8	118.2 (2)	H18B—C18—H18C	109.5
			
C9—C1—C2—C3	1.2 (4)	N1—C8—C9—C1	179.8 (2)
Br1—C1—C2—C3	−178.45 (18)	C4—C8—C9—C1	−0.2 (3)
C1—C2—C3—C4	−0.8 (4)	N1—C8—C9—O1	−3.4 (3)
C1—C2—C3—Br2	−179.18 (17)	C4—C8—C9—O1	176.6 (2)
C2—C3—C4—C5	178.9 (2)	C9—O1—C10—C11	−138.9 (2)
Br2—C3—C4—C5	−2.8 (3)	C12—N2—C11—O2	−1.6 (4)
C2—C3—C4—C8	0.0 (3)	C12—N2—C11—C10	175.2 (2)
Br2—C3—C4—C8	178.33 (17)	O1—C10—C11—O2	−149.5 (2)
C3—C4—C5—C6	179.9 (2)	O1—C10—C11—N2	33.5 (3)
C8—C4—C5—C6	−1.2 (3)	C11—N2—C12—C13	−2.4 (4)
C4—C5—C6—C7	0.7 (4)	C11—N2—C12—C17	177.4 (2)
C8—N1—C7—C6	0.9 (3)	C17—C12—C13—C14	1.0 (4)
C5—C6—C7—N1	−0.6 (4)	N2—C12—C13—C14	−179.2 (2)
C7—N1—C8—C4	−1.4 (3)	C12—C13—C14—C15	0.1 (4)
C7—N1—C8—C9	178.6 (2)	C13—C14—C15—C16	−1.1 (4)
C5—C4—C8—N1	1.6 (3)	C14—C15—C16—C17	1.1 (4)
C3—C4—C8—N1	−179.4 (2)	C18—O3—C17—C16	10.3 (3)
C5—C4—C8—C9	−178.5 (2)	C18—O3—C17—C12	−169.3 (2)
C3—C4—C8—C9	0.5 (3)	C15—C16—C17—O3	−179.5 (2)
C2—C1—C9—O1	−177.3 (2)	C15—C16—C17—C12	0.0 (4)
Br1—C1—C9—O1	2.3 (3)	C13—C12—C17—O3	178.5 (2)
C2—C1—C9—C8	−0.7 (3)	N2—C12—C17—O3	−1.3 (3)
Br1—C1—C9—C8	178.94 (17)	C13—C12—C17—C16	−1.1 (4)
C10—O1—C9—C1	−90.4 (3)	N2—C12—C17—C16	179.1 (2)
C10—O1—C9—C8	92.9 (2)		

### Hydrogen-bond geometry (Å, °)

**Table:**

*D*—H···*A*	*D*—H	H···*A*	*D*···*A*	*D*—H···*A*
N2—H2A···N1	0.90 (1)	2.24 (1)	3.065 (3)	153 (1)
C18—H18C···O2^i^	0.96	2.53	3.342 (3)	142

Symmetry codes:  (i) *x*, *y*−1, *z*.
